# Perceived HBV‐Related Stigma Is Associated With Lower Antiviral Medication Adherence in Patients With Chronic Hepatitis B

**DOI:** 10.1111/jvh.70010

**Published:** 2025-02-15

**Authors:** Hee‐Soon Juon, Daniel Yang, Cindy Xin Fang, Hie‐Won Hann, Ho Bae, Mimi Chang, Ann C. Klassen

**Affiliations:** ^1^ Department of Medical Oncology Thomas Jefferson University Philadelphia Pennsylvania USA; ^2^ Liver Disease Prevention Center Thomas Jefferson University Hospital Philadelphia Pennsylvania USA; ^3^ Asian Pacific Liver Center Coalition of Inclusive Medicine Los Angeles California USA; ^4^ Dornsife School of Public Health Drexel University Philadelphia Pennsylvania USA

**Keywords:** Asian Americans, CHB patients, Korean Americans, medication adherence, MMAS‐8

## Abstract

Medication nonadherence among patients with chronic hepatitis B (CHB) can lead to severe liver disease progression, including liver cirrhosis and hepatocellular carcinoma (HCC). Yet the factors that influence adherence in high‐risk groups, like Korean Americans, remain unclear. Thus, this study explored the psychosocial and clinical factors affecting medication adherence in CHB patients. A cohort of 365 Korean American patients with CHB from two clinics in Philadelphia and Los Angeles was studied. The 8‐item Morisky Medication Adherence Scale (MMAS‐8) gauged their adherence to antiviral medication. Using descriptive and multivariable logistic regression analyses, we identified factors associated with MMAS‐8 scores. Of the participants, 78% were undergoing antiviral therapy, with over two‐thirds (69%) reporting medium to high adherence levels. The multivariable logistic regression analysis revealed that age, knowledge of sequalae of CHB, perceived HBV stigma and possession of pharmacy plan were associated with medication adherence. Older participants had higher medication adherence than younger. High knowledge of sequalae of CHB and low perceived HBV stigma were associated with higher medication adherence. Having pharmacy plans was also associated with higher medication adherence to antiviral therapy. These findings highlight the critical role of person‐related factors (e.g., knowledge and stigma) and healthcare factors in medication adherence. Future research should focus on developing targeted educational interventions focusing on personal factors to improve medication adherence among Korean American patients with CHB.

## 1 Introduction

Hepatitis B virus (HBV) infection is one of the leading causes of death among communicable diseases [1]. According to the World Health Organization (WHO) 2022 global report, approximately 254 million people were living with chronic hepatitis B (CHB) infection, with an estimated 1.2 million new infections reported annually [1, 2]. CHB infections can lead to severe long‐term complications, including cirrhosis and hepatocellular carcinoma (HCC), which are the primary causes of HBV‐related morbidity and mortality [1]. In the United States, CHB disproportionately affects Asian Americans, with the rate of new cases among non‐Hispanic Asian/Pacific Islanders (20.1 cases per 100,000) being 10.2 times higher than among non‐Hispanic Whites (1.8 cases per 100,000) [3, 4]. The mortality rate due to HBV infection is also the highest among non‐Hispanic Asian/Pacific Islanders (2.30 deaths per 100,000), 8.5 times greater than among non‐Hispanic Whites (0.27 deaths per 100,000) [3, 5]. Specifically, Korean Americans in particular face elevated liver cancer mortality rates, with 33.9 deaths per 100,000 males, in stark contrast to 6.8 among non‐Hispanic white males [6]. If their disease is left unmanaged or untreated, 25% of patients with CHB may develop HCC [7, 8].

Medication nonadherence may lead to treatment failure and drug resistance [9]. Antiviral therapy for CHB effectively reduces HBV viral load and lowers the risk of developing severe liver‐related complications and liver cancer [10, 11, 12]. A 5‐year longitudinal study of CHB patients receiving entecavir (ETV) treatment identified adherence as a significant predictor of liver‐related complications in multivariate analysis. Compared with the optimal adherence group (> 90%), patients with poor adherence (< 70%) exhibited a 2.9‐fold higher risk of developing HCC and cirrhotic complications, a 14.3‐fold increase in liver‐related mortality, and a 5‐fold increase in all‐cause mortality [13]. A meta‐analysis indicated that the average medication adherence among CHB patients treated with antiviral therapies, such as tenofovir alafenamide (TAF), tenofovir disoproxil fumarate (TDF), and entecavir (ETV), is approximately 74.6%, significantly lower than the optimal adherence (i.e., 95%) recommended for effective disease management [13]. This finding underscores the need for systematic medication adherence assessments and evidence‐based strategies in clinical practice [14, 15, 16].

Most studies assessing CHB medication adherence have utilised methods such as pill counts, pharmacy refill data, electronic medication monitoring or general self‐report questionnaires [14, 17]. However, these approaches often fail to capture behavioural and attitudinal dimensions of medication‐taking behaviour. The Morisky Medication Adherence Scale (MMAS‐8) provides a more comprehensive and structured assessment to address these gaps. MMAS‐8 is a widely used tool for assessing nonadherence and overall treatment success among patients with chronic conditions such as diabetes, hypertension and osteoporosis [18]. A systematic review of studies found that the MMAS‐8 had strong psychometric properties, including good reliability and validity for measuring medication adherence [18]. MMAS‐8 has also demonstrated a strong correlation with several important long‐term outcomes, including the proportion of patients managing their health condition and reduced frequency of emergency department visits [19, 20]. These findings highlight its potential to stratify high‐risk nonadherent patients and identify possible individual barriers to adherence. Investigative efforts utilising the MMAS‐8 have shed light on antiviral medication adherence levels and influential factors among CHB patients within Chinese, Chinese American and Vietnamese American communities, revealing adherence rates ranging from 48.8% to 66% [21, 22]. Factors associated with medication adherence were acculturation (living more than 10 years in the United States) and an in‐depth understanding of HBV [21]. Conversely, younger age, recent treatment initiation and female gender were identified as predictors for nonadherence [21, 23].

Given the critical role of medication adherence in improving CHB patient outcomes, it is crucial to identify the underlying factors that contribute to medication adherence in Asian American Communities. The WHO proposes a comprehensive framework for studying underlying factors influencing medication adherence, determined by five key dimensions: (1) social and economic factors (e.g., education, income, language, and culture), (2) healthcare system factors (e.g., healthcare access, insurance plan coverage, provision of training, communication between patients and provider), (3) patient factors (e.g., age, gender, knowledge, health beliefs, support, and stigma). (4) medication factors (e.g., medication regimen, and medication effects), and (5) condition factors (e.g., disease characteristics, disease control) [24]. Using this framework, a systematic review identified the top three dimensions influencing medication adherence for adults undergoing treatment for chronic, communicable conditions: patient‐related, social/economic, and healthcare system‐related [24]. However, this conclusion was primarily based on medication adherence research on HIV and tuberculosis, with limited studies on CHB, despite its increasingly high morbidity and mortality [2, 25].

Furthermore, there is limited research specifically examining medication adherence factors for CHB in Asian American populations, particularly the Korean American community. Therefore, our study aims to bridge this gap in current research by (1) assessing CHB medication adherence using MMAS‐8 in Korean Americans and (2) identifying and categorising factors influencing medication adherence in Korean Americans based on WHO's adaptable framework. In this study, two factors are held constant across our study population: The condition (e.g., CHB) is the same and the medications are typically the same (e.g., antiviral medication). Thus, we will focus on social and economic factors, healthcare system‐related factors, and person‐related factors among Korean American patients with CHB currently taking antiviral therapy.

## 2 Methods

### 2.1 Study Design

The design and data collection procedures for this prospective cohort study, Happy Life and Health Liver (HL2), have been previously described in detail [26]. The cohort was recruited from two clinical sites, Thomas Jefferson University Hospital and the Asian Pacific Liver Center, following medical chart review. We utilised baseline survey data from 365 participants who completed baseline activities from August 2022 to January 2023. This study received approval from the Thomas Jefferson University Institutional Review Board.

#### 2.1.1 Baseline Survey

After providing written consent, 365 CHB respondents completed the baseline activities, including hair sampling. Given the choice of language for the materials, 82% (*n* = 299) selected Korean, and 18% (*n* = 66) chose English. Most respondents (83%) opted for self‐administered modality (63% using an iPad on site; 26% using a remote link; and 11% completing a paper copy). Due to reading difficulties or discomfort with using an iPad, 17% of participants requested the questionnaire be administered by an interviewer. The baseline survey took approximately 40 min to complete, and participants received a $50 gift card for their involvement.

As a part of a retrospective and prospective longitudinal study, the reported data included an analysis of the baseline assessments. The baseline survey encompassed sociodemographic data, acculturation, mental health status, access to healthcare, medication adherence, HBV stigma, knowledge of HBV transmission, sequalae of CHB and treatment.

### 2.2 Measures

#### 2.2.1 Outcome Measure

HBV medication adherence was assessed using MMAS‐8 [27]. This scale examines adherence by posing eight questions, seven with binary options (yes/no), and the eighth question with possible five responses ranging from never (0) to all the time (4). In line with prior research, the scores from these eight questions were summed to create the construct of MMAS‐8 and then divided into binary indicators. A score between 0 and 5 indicated low adherence, while a score of 6–8 signified medium/high adherence (i.e., good adherence).

*Patient factors* encompassed age, gender (1 = male; 2 = female), knowledge of HBV transmission, sequelae of CHB and perceived HBV stigma, and social support. Knowledge of HBV transmission was measured by asking 10 potential modes of HBV transmission (e.g., from infected mother to child during childbirth; by sharing a razor with an infected person) [28]. Each correctly answered question was given a point; scores were summed, and the final score ranged from 0 to 10. Knowledge of Sequelae of CHB was measured by asking seven questions about perceived consequences of CHB (e.g., people with HBV are infected for life; hepatitis B infection cannot be cured, but the disease can be managed) [26, 28]. Each correctly answered question was given a point; scores were summed, and the final knowledge score ranged from 0 to 7. In this analysis, it was categorised into two (L = 0–4; High = 5–7). Perceived HBV stigma was measured by the Hepatitis B quality of life (HBQOL) Instrument—stigma subscale [29]. Six questions have five‐point Likert scale (never [= 0] to all the times [= 4]). The sum of stigma ranged from 0 to 24. It was categorised into 3 based on tertiles (L = 0–2; M = 3–5; H = 6+). Cronbach's alpha for the current study was 0.93. Social support was measured with an 8‐item modified version of the Medical Outcomes Study Social Support (mMOS‐SS) scale [30], measuring five levels of availability (none of the time [= 1] to all the time [= 5]) of key types of social support, from instrumental to emotional supports (e.g., help if confined to bed; help with daily chores; advice on dealing with an issue; providing love/feeling wanted). The sum of social support ranged from 8 to 40. Cronbach's alpha for the current study was 0.96.

*Sociodemographic factors* were also considered, including education (1 = less than high school; 2 = high school graduate; 3 = college graduate or higher).

*Healthcare system factors* were measured by possession of pharmacy plan (0 = no; 1 = yes).

### 2.3 Statistical Analysis

Descriptive and analytic statistical methods were employed to present the findings of this study. Survey responses were analysed using frequency and crosstabulation through chi‐squared analysis for categorical variables and Student's *t*‐test for continuous variables. Both bivariate and multivariate logistic regression were utilised to identify factors contributing to medication adherence. The multicollinearity among factors were checked: Since education and income were highly correlated (*r* = 0.44, *p* < 0.001), only education was included. Stata version 17 was the software used for analysis.

## 3 Results

Table 1 outlines the characteristics of the cohort. The average age of 365 participants was 60.1 years (range 19–84, SD 10.7). The cohort was 56% male, with 97% born in South Korea. Approximately 56% had completed college, and a third reported poor or no English proficiency. A majority (81%) were married or living with a partner and about two‐thirds were employed. The vast majority (92%) had some form of health insurance. The average time since HBV diagnosis was 26.9 years (range 5–52, SD 10.3), and 58% had family history of HBV infection.

**TABLE 1:** Characteristics of Korean American CHB patients (*n* = 365), 2021–2022.

Variables (%)	*N* (%)
Sociodemographic	
Age (mean ± SD, range)	60.09 ± 10.74 (19–84)
Gender	
Men	203 (55.6%)
Women	162 (44.4%)
Education	
< High school	23 (6.4%)
High school graduate+	136 (37.6%)
College graduate	133 (36.7%)
Graduate/professional school	70 (19.3%)
Spoken English proficiency	
Fluent/well	96 (26.5%)
So so	146 (40.2%)
Poor/not at all	121 (33.3%)
Marital status	
Married	295 (80.8%)
Not married (divorce/widow/separate/single)	70 (19.2%)
Employment (= yes)	227 (62.4%)
Having health insurance (= yes)	337 (92.3%)
Time since HBV diagnosis (year), (mean ± SD, range)	26.97 ± 10.33 (5–52)
Family history of HBV infection (= yes)	213 (58.4%)
Knowledge of HBV transmission (mean ± SD, range)	6.30 ± 2.17 (0–10)
Sequalae of CHB (mean ± SD, range)	4.92 ± 1.32 (0–10)
Perceived HBV stigma (mean ± SD, range)	5.38 ± 5.69 (0–24)
Social support (mean ± SD, range)	29.50 ± 8.60 (8–40)
Outcome	
Taking antiviral medication (= yes)	283 (77.5%)

As the current analysis concentrated on factors of medication adherence at baseline, participants not currently on HBV medication, as indicated by the medical chart review (*n* = 82), were omitted, resulting in an analytic sample of 283 participants (78%). Most common antiviral medications were tenofovir alafenamide (TAF: 32.5%) and tenofovir disoproxil fumarate (TDF: 30.4%). About one‐fifth (22.3%) were taking lamivudine followed by entecavir (ETV: 14%) (see Table 2).

**TABLE 2:** Type of antiviral medication among Korean American CHB patients (*n* = 283).

Medication	*N* (%)
Tenofovir alafenamide (TAF)	92 (32.5%)
Tenofovir disopoxil fumerate (TDF)	86 (30.4%)
Lamivudine	63 (22.3%)
Entecavir (ETV)	39 (13.7%)
Adefovir	1 (0.3%)
Others	2 (0.7%)

Table 3 presents the results of bivariate and multivariable logistic regression analyses. The bivariate analysis identified several factors associated with medication adherence, including sequalae of CHB, social support, perceived HBV stigma and possession of a pharmacy plan. In the multivariable logistic regression analysis, age, sequalae of CHB, perceived HBV stigma and possession of pharmacy plan remained significant factors at *p* value < 0.05: older had higher medication adherence than younger (aOR = 1.04, 95% CI 1.01–1.07). Those with high sequelae of CHB were more likely to have medication adherence than those with low scores (aOR = 2.04, 95% CI = 1.11–3.78). Those with medium perceived HBV stigma had lower medication adherence than those with low stigma (aOR = 0.47, 95% CI 0.24–0.91). Those who had pharmacy plans were more likely to adhere antiviral therapy (aOR = 1.94, 95% CI 1.01–3.74). Social support was marginally associated with medication adherence, showing higher social support, higher medication adherence.

**TABLE 3:** Factors contributing to medication adherence among 283 CHB patients who take antiviral medication.

Variables	Unadjusted OR (95% CI)	Adjusted OR (95% CI) (*n* = 278)
I. Patient‐related factors		
Age	1.02 (0.99–1.05)^+^	1.04 (1.01–1.07)*
Gender		
Males	1.00	1.00
Females	0.80 (0.48–1.34)	0.75 (0.43–1.33)
Knowledge of HBV transmission	1.02 (0.91–1.15)	1.11 (0.96–1.27)
Sequalae of CHB		
Low	1.00	1.00
Medium/high	1.96 (1.10–3.51)*	2.04 (1.11–3.78)*
Social supports	1.03 (1.01–1.06)*	1.03 (0.99–1.06)^+^
Perceived HBV stigma		
Low	1.00	1.00
Medium	0.50 (0.27–0.94)*	0.47 (0.24–0.91)*
High	0.63 (0.34–1.17)	0.73 (0.38–1.39)
II. Sociodemographic factors		
Education		
< High school	1.00	1.00
High school graduate+	1.62 (0.59–4.46)	1.71 (0.61–4.79)
College graduate+	2.11 (0.78–5.72)	2.33 (0.83–6.54)
III. Healthcare system factor		
Having pharmacy plan		
No	1.00	1.00
Yes	1.90 (1.01–3.59)*	1.94 (1.01–3.74)*

^+^ *p* < 0.10.

* *p* < 0.05.

## 4 Discussion

This study identified factors contributing to self‐reported antiviral medication adherence in a sample of Korean American patients with CHB. A relatively high percentage of patients (69%) reported good medication adherence as measured by the MMAS‐8, comparable to the 66% adherence rate found in previous studies with Chinese and Vietnamese American patients with CHB [22]. A systematic review indicated considerable variability in medication adherence rates across different patient populations, with discrepancies observed between Asian American patients (66%) and Chinese residents (49%) [21, 22, 23]. This likely reflects the unique economic and health resource disparities between the United States and China.

Consistent with previous research [21], our study observed that older CHB patients had higher rates of medication adherence. One reason could be that older persons may be more accepting of taking daily medications than younger persons and may already do so for other common diseases of aging, such as hypertension. A study conducted in Korea found that adherence to antihypertensive medications increased with age until participants reached 69 years, then began to decline [31]. This could be attributed to increased health awareness and potentially more severe disease presentations in older individuals [19]. We found that younger patients experience higher levels of perceived HBV‐related stigma (*r* = −0.10, *p* = 0.058). This increased stigma may contribute to their lower adherence to antiviral medication therapy.

Our findings align with previous research, suggesting that an in‐depth understanding of CHB is a critical factor in ensuring good medication adherence [32, 33]. Individuals with higher knowledge of sequelae of CHB may possess greater knowledge of the risks associated with nonadherence, such as virological failure and disease progression risks like liver cirrhosis or cancer [23]. Health education aimed at increasing understanding of the importance and consequences of nonadherence to antiviral therapy among CHB patients is essential. This knowledge may empower them to achieve lifetime CHB management by adherence to antiviral therapy.

Perceived HBV impacts disease management and the physical and mental health of patients [26, 34]. In this study, we found that perceived HBV stigma was associated with medication adherence. Notably, patients with high perceived HBV stigma exhibited significantly lower adherence to antiviral medication. Lack of knowledge about the consequences of CHB and misconceptions of HBV transmission are drivers of HBV‐related stigma [35, 36].

Consistent with previous studies of chronic communicable conditions such as HIV [37, 38], access to health care is another contributing factor for medication adherence. In this study, most CHB patients (92%) had some type of health insurance, which did not predict adherence to antiviral medication due to lack of variability. However, pharmacy plan (81%) was a far more important contributing factor to medication adherence.

Analysing predictors of antiviral medication adherence in the CHB patient population is important, as only a small percentage achieve a “functional cure”—the clearance of HBsAg—using current nucleotide analogue treatments, which were the medications administered to patients in our study. While nucleotide analogues are highly effective at suppressing HBV DNA [39], the annual rate of HBsAg clearance is only about 1% [40]. A systemic review and meta‐analysis further indicates that seroclearance of HBV infection mainly occurs in patients with less active disease [41]. As a result, the majority of CHB patients require lifelong adherence to antiviral regimens to prevent liver disease progression and the development of hepatocellular carcinoma [41]. Identifying and addressing potential barriers to adherence is therefore critical for optimising the outcomes of antiviral therapy, particularly nucleotide analogues. Even if those patients with functional cure of hepatitis B do not need medication, they will be encouraged to continue regular monitoring with their doctor through blood tests to ensure the virus remains undetectable.

In addition, WHO updated its HBV treatment guidelines in 2024 [42], expanding treatment criteria to a larger population, including young adults, adolescents, and adding antiviral prophylaxis for pregnant women. Hence, there is a growing need to ensure adherence to antiviral medications across a larger, more diverse patient population. By identifying key predictors of adherence, our analysis provides valuable insights that can support the successful implementation of these expanded treatment guidelines.

One of the strengths of this study was identifying factors to adherence to antiviral medication using the conceptual framework. The specific factors driving low medication adherence among high‐risk CHB patients, particularly Korean Americans, have not been identified. We examined the contributing factors to adherence of antiviral therapy in a population of Korean American patients with CHB based on an adaptable framework.

Another strength of this study is that the population was specific to Korean American patients with CHB receiving care from Korean American hepatologists. Previous studies about HBV among Korean Americans have been limited to Korean Americans in the community or those receiving care in general healthcare system [43, 44]. To our knowledge, this is the first study to identify factors related to medication adherence using MMAS‐8.

This study also had several limitations. Recruiting participants from only two clinical sites in Philadelphia and Los Angeles restricts the generalisability of the results to all Korean American patients with CHB in the United States. In addition, reliance on self‐reported data raises concerns about potential recall bias and inaccuracies.

In conclusion, these results suggest that culturally tailored educational interventions that address personal—and healthcare system—factors are essential for improving medication adherence among Korean Americans living with CHB in the United States. Improving patient awareness through educational intervention, medication management, and patient navigation may promote better antiviral medication adherence, especially among younger patients and those with low knowledge of HBV sequelae and high perceived HBV stigma. Given the recent WHO guidelines that broaden treatment recommendations for antiviral therapy to a larger proportion of HBV‐infected individuals, addressing medication adherence is crucial [42].

## Ethics Statement

All procedures performed in these studies were approved by the Institutional Review Board of Thomas Jefferson University.

## Consent

Informed consent was obtained from all individual participants included in the study.

## Conflicts of Interest

The authors declare no conflicts of interest.

## Data Availability

The data that support the findings of this study are available from the corresponding author upon reasonable request.

## References

[1] “Hepatitis B,” accessed November 17, 2024, https://www.who.int/news‐room/fact‐sheets/detail/hepatitis‐b.

[2] “Global Hepatitis Report 2024: Action for Access in Low‐ and Middle‐Income Countries,” accessed November 17, 2024, https://www.who.int/publications/i/item/9789240091672.

[3] “2022 Hepatitis B | Viral Hepatitis Surveillance Report” (CDC, 2024), https://www.cdc.gov/hepatitis/statistics/2022surveillance/hepatitis‐b.htm.

[4] “2022 Newly Reported Chronic Hepatitis B by Demographics” (CDC, 2024), https://www.cdc.gov/hepatitis/statistics/2022surveillance/hepatitis‐b/table‐2.6.htm.

[5] “2018–2022 Numbers & Rates of Deaths With Hepatitis B Listed as Cause by Demographics” (CDC, 2024), https://www.cdc.gov/hepatitis/statistics/2022surveillance/hepatitis‐b/table‐2.8.htm.

[6] M. H. Le , Y. H. Yeo , R. Cheung , L. Henry , A. S. Lok , and M. H. Nguyen , “Chronic Hepatitis B Prevalence Among Foreign‐Born and U.S.‐Born Adults in the United States, 1999‐2016,” Hepatology 71, no. 2 (2020): 431–443, 10.1002/hep.30831. 31228279

[7] C. L. Lin and J. H. Kao , “Development of Hepatocellular Carcinoma in Treated and Untreated Patients With Chronic Hepatitis B Virus Infection,” Clinical and Molecular Hepatology 29, no. 3 (2023): 605–622, 10.3350/cmh.2022.0342. 36788759 PMC10366811

[8] L. R. Roberts , “Untreated Chronic Hepatitis B Is Associated With a Higher Risk of Extrahepatic Malignancies,” Journal of Clinical Oncology 40, no. 29 (2022): 3357–3360, 10.1200/JCO.22.01051. 35853191

[9] J.‐M. Pawlotsky , G. Dusheiko , A. Hatzakis , et al., “Virologic Monitoring of Hepatitis B Virus Therapy in Clinical Trials and Practice: Recommendations for a Standardized Approach,” Gastroenterology 134, no. 2 (2008): 405–415, 10.1053/j.gastro.2007.11.036. 18242209 PMC2676233

[10] R. N. Chien and Y. F. Liaw , “Current Trend in Antiviral Therapy for Chronic Hepatitis B,” Viruses 14, no. 2 (2022): 434, 10.3390/v14020434. 35216027 PMC8877417

[11] N. L. Allard , J. H. MacLachlan , A. Dev , et al., “Adherence in Chronic Hepatitis B: Associations Between Medication Possession Ratio and Adverse Viral Outcomes,” BMC Gastroenterology 20, no. 1 (2020): 140, 10.1186/s12876-020-01219-w. 32381025 PMC7203797

[12] N. B. Ha , N. B. Ha , R. T. Garcia , et al., “Medication Nonadherence With Long‐Term Management of Patients With Hepatitis B e Antigen‐Negative Chronic Hepatitis B,” Digestive Diseases and Sciences 56, no. 8 (2011): 2423–2431, 10.1007/s10620-011-1610-5. 21327918

[13] J. W. Shin , S. W. Jung , S. B. Lee , et al., “Medication Nonadherence Increases Hepatocellular Carcinoma, Cirrhotic Complications, and Mortality in Chronic Hepatitis B Patients Treated With Entecavir,” American Journal of Gastroenterology 113, no. 7 (2018): 998–1008, 10.1038/s41395-018-0093-9. 29880971

[14] N. Ford , R. Scourse , M. Lemoine , et al., “Adherence to Nucleos(t)ide Analogue Therapies for Chronic Hepatitis B Infection: A Systematic Review and Meta‐Analysis,” Hepatology Communications 2, no. 10 (2018): 1160–1167, 10.1002/hep4.1247. 30288470 PMC6167073

[15] L. Zhang , J. Tian , D. Xu , Y. Liu , and Z. Zhang , “Trajectory and Predictors of Adherence to Nucleos(t)ide Analogues Medication Among Patients With Chronic Hepatitis B,” Heliyon 10, no. 19 (2024): e38485, 10.1016/j.heliyon.2024.e38485. 39391516 PMC11466648

[16] K. P. Chai , V. Saxena , S. Seo , et al., “A Chronic Hepatitis B Identification and Surveillance Program Improves Care in an Integrated Health Plan,” American Journal of Gastroenterology (2024), 10.14309/ajg.0000000000003116. 39364887

[17] L. Osterberg and T. Blaschke , “Adherence to Medication,” New England Journal of Medicine 353 (2005): 487–497, 10.1056/NEJMra050100. 16079372

[18] S. J. Moon , W. Y. Lee , J. S. Hwang , Y. P. Hong , and D. E. Morisky , “Accuracy of a Screening Tool for Medication Adherence: A Systematic Review and Meta‐Analysis of the Morisky Medication Adherence Scale‐8,” PLoS One 12, no. 11 (2017): e0187139, 10.1371/journal.pone.0187139. 29095870 PMC5667769

[19] B. Uchmanowicz , E. A. Jankowska , I. Uchmanowicz , and D. E. Morisky , “Self‐Reported Medication Adherence Measured With Morisky Medication Adherence Scales and Its Determinants in Hypertensive Patients Aged ≥60 Years: A Systematic Review and Meta‐Analysis,” Frontiers in Pharmacology 10 (2019): 168, 10.3389/fphar.2019.00168. 30930769 PMC6425867

[20] W. Y. Lam and P. Fresco , “Medication Adherence Measures: An Overview,” BioMed Research International 2015, no. 1 (2015): 217047, 10.1155/2015/217047. 26539470 PMC4619779

[21] K. Xu , L. M. Liu , P. A. Farazi , et al., “Adherence and Perceived Barriers to Oral Antiviral Therapy for Chronic Hepatitis B,” Global Health Action 11, no. 1 (2018): 1433987, 10.1080/16549716.2018.1433987. 29447614 PMC5827725

[22] A. Bhimla , L. Zhu , W. Lu , et al., “Factors Associated With Hepatitis B Medication Adherence and Persistence Among Underserved Chinese and Vietnamese Americans,” Journal of Clinical Medicine 11, no. 3 (2022): 870, 10.3390/jcm11030870. 35160319 PMC8837110

[23] F. I. Lieveld , L. G. van Vlerken , P. D. Siersema , and K. J. van Erpecum , “Patient Adherence to Antiviral Treatment for Chronic Hepatitis B and C: A Systematic Review,” Annals of Hepatology 12, no. 3 (2013): 380–391, 10.1016/S1665-2681(19)31000-2. 23619254

[24] World Health Organization , “Adherence to Long‐Term Therapies: Evidence for Action” (World Health Organization, 2003), https://iris.who.int/handle/10665/42682.

[25] K. Q. E. Peh , Y. H. Kwan , H. Goh , et al., “An Adaptable Framework for Factors Contributing to Medication Adherence: Results From a Systematic Review of 102 Conceptual Frameworks,” Journal of General Internal Medicine 36, no. 9 (2021): 2784–2795, 10.1007/s11606-021-06648-1. 33660211 PMC8390603

[26] J. G. Katcher , A. C. Klassen , H. W. Hann , M. Chang , and H. S. Juon , “Racial Discrimination, Knowledge, and Health Outcomes: The Mediating Role of Hepatitis B‐Related Stigma Among Patients With Chronic Hepatitis B,” Journal of Viral Hepatitis 31 (2024): 248–254, 10.1111/jvh.13932. 38409935 PMC11023788

[27] D. E. Morisky , A. Ang , M. Krousel‐Wood , and H. J. Ward , “Predictive Validity of a Medication Adherence Measure in an Outpatient Setting,” Journal of Clinical Hypertension 10, no. 5 (2008): 348–354, 10.1111/j.1751-7176.2008.07572.x. 18453793 PMC2562622

[28] H. S. Juon and B. J. Park , “Effectiveness of a Culturally Integrated Liver Cancer Education in Improving HBV Knowledge Among Asian Americans,” Preventive Medicine 56, no. 1 (2013): 53–58, 10.1016/j.ypmed.2012.11.003. 23159302 PMC3540148

[29] B. M. Spiegel , R. Bolus , S. Han , et al., “Development and Validation of a Disease‐Targeted Quality of Life Instrument in Chronic Hepatitis B: The Hepatitis B Quality of Life Instrument, Version 1.0,” Hepatology 46, no. 1 (2007): 113–121, 10.1002/hep.21692. 17596882

[30] A. Moser , A. E. Stuck , R. A. Silliman , P. A. Ganz , and K. M. Clough‐Gorr , “The Eight‐Item Modified Medical Outcomes Study Social Support Survey: Psychometric Evaluation Showed Excellent Performance,” Journal of Clinical Epidemiology 65, no. 10 (2012): 1107–1116, 10.1016/j.jclinepi.2012.04.007. 22818947 PMC4119888

[31] S. J. Kim , O. D. Kwon , E. B. Han , et al., “Impact of Number of Medications and Age on Adherence to Antihypertensive Medications,” Medicine 98, no. 49 (2019): e17825, 10.1097/MD.0000000000017825. 31804305 PMC6919523

[32] R. Shiraly , A. Khani Jeihooni , and R. Bakhshizadeh Shirazi , “Perception of Risk of Hypertension Related Complications and Adherence to Antihypertensive Drugs: A Primary Healthcare Based Cross‐Sectional Study,” BMC Primary Care 23, no. 1 (2022): 303, 10.1186/s12875-022-01918-1. 36443657 PMC9706951

[33] P. Shakya , A. Shrestha , B. M. Karmacharya , D. E. Morisky , and B. E. Kulseng , “Factors Associated With Medication Adherence Among Patients With Type 2 Diabetes Mellitus: A Hospital‐Based Cross‐Sectional Study in Nepal,” International Journal of Environmental Research and Public Health 20, no. 2 (2023): 1537, 10.3390/ijerph20021537. 36674292 PMC9866714

[34] D. Li , T. Tang , M. Patterson , M. Ho , J. Heathcote , and H. Shah , “The Impact of Hepatitis B Knowledge and Stigma on Screening in Canadian Chinese Persons,” Canadian Journal of Gastroenterology 26, no. 9 (2012): 597–602. 22993729 10.1155/2012/705094 PMC3441165

[35] D. Jin , L. Brener , and C. Treloar , “Hepatitis B‐Related Stigma Among Chinese Immigrants Living With Hepatitis B Virus in Australia: A Qualitative Study,” Health & Social Care in the Community 30, no. 6 (2022): e5602–e5611, 10.1111/hsc.13986. 36068665 PMC10086810

[36] L. Valizadeh , V. Zamanzadeh , M. Bayani , and A. Zabihi , “The Social Stigma Experience in Patients With Hepatitis B Infection: A Qualitative Study,” Gastroenterology Nursing 40, no. 2 (2017): 143–150, 10.1097/SGA.0000000000000223. 28362662

[37] A. Dubov , F. L. Altice , and L. Fraenkel , “An Information‐Motivation‐Behavioral Skills Model of PrEP Uptake,” AIDS and Behavior 22, no. 11 (2018): 3603–3616, 10.1007/s10461-018-2095-4. 29557540 PMC9626245

[38] K. B. Jacobson , L. Niccolai , N. Mtungwa , A. P. Moll , and S. V. Shenoi , “It's About My Life: Facilitators of and Barriers to Isoniazid Preventive Therapy Completion Among People Living With HIV in Rural South Africa,” AIDS Care 29, no. 7 (2017): 936–942, 10.1080/09540121.2017.1283390. 28147705 PMC5545149

[39] J. Adam , “Gehring, New Treatments to Reach Functional Cure: Rationale and Challenges for Emerging Immune‐Based Therapies,” Best Practice & Research Clinical Gastroenterology 31, no. 3 (2017): 337–345, 10.1016/j.bpg.2017.05.004. 28774416

[40] B. V. Chihota , G. Wandeler , R. Chilengi , et al., “High Rates of Hepatitis B Virus (HBV) Functional Cure Among Human Immunodeficiency Virus‐HBV Coinfected Patients on Antiretroviral Therapy in Zambia,” Journal of Infectious Diseases 221, no. 2 (2020): 218–222, 10.1093/infdis/jiz450. 31613956 PMC7184905

[41] Y. H. Yeo , H. J. Ho , H. I. Yang , et al., “Factors Associated With Rates of HBsAg Seroclearance in Adults With Chronic HBV Infection: A Systematic Review and Meta‐Analysis,” Gastroenterology 156, no. 3 (2019): 635–646, 10.1053/j.gastro.2018.10.027. 30342034

[42] Guidelines for the Prevention, Diagnosis, Care and Treatment for People With Chronic Hepatitis B Infection (World Health Organization, 2024). 40424433

[43] S. Hyun , O. Ko , S. Kim , and W. R. Ventura , “Sociocultural Barriers to Hepatitis B Health Literacy in an Immigrant Population: A Focus Group Study in Korean Americans,” BMC Public Health 21, no. 1 (2021): 404, 10.1186/s12889-021-10441-4. 33632203 PMC7908637

[44] S. Hyun , S. Lee , W. R. Ventura , and J. McMenamin , “Knowledge, Awareness, and Prevention of Hepatitis B Virus Infection Among Korean American Parents,” Journal of Immigrant and Minority Health 20, no. 4 (2018): 943–950, 10.1007/s10903-017-0609-1. 28639095 PMC6061079

